# Influence of sex and stress exposure across the lifespan on endophenotypes of depression: focus on behavior, glucocorticoids, and hippocampus

**DOI:** 10.3389/fnins.2014.00420

**Published:** 2015-01-06

**Authors:** Aarthi R. Gobinath, Rand Mahmoud, Liisa A.M. Galea

**Affiliations:** ^1^Program in Neuroscience, Centre for Brain Health, University of British ColumbiaVancouver, BC, Canada; ^2^Department of Psychology, University of British ColumbiaVancouver, BC, Canada

**Keywords:** sex differences, depression, HPA axis, hippocampal neurogenesis, adolescence

## Abstract

Sex differences exist in vulnerability, symptoms, and treatment of many neuropsychiatric disorders. In this review, we discuss both preclinical and clinical research that investigates how sex influences depression endophenotypes at the behavioral, neuroendocrine, and neural levels across the lifespan. Chronic exposure to stress is a risk factor for depression and we discuss how stress during the prenatal, postnatal, and adolescent periods differentially affects males and females depending on the method of stress and metric examined. Given that the integrity of the hippocampus is compromised in depression, we specifically focus on sex differences in how hippocampal plasticity is affected by stress and depression across the lifespan. In addition, we examine how female physiology predisposes depression in adulthood, specifically in postpartum and perimenopausal periods. Finally, we discuss the underrepresentation of women in both preclinical and clinical research and how this limits our understanding of sex differences in vulnerability, presentation, and treatment of depression.

## Introduction

### Sex differences in depression

There are a number of sex differences in incidence, manifestation, symptoms, and treatment efficacy of neuropsychiatric disorders, however often these sex differences are ignored in the literature (Cahill, 2006). Even at the cellular levels, chromosomal influences (XX or XY genotype) can have profound effects on the cellular activity of every cell in the body, including neurons (Penaloza et al., 2009; Straface et al., 2012). Thus, it is curious that such a fundamental aspect of cellular function and physiology is largely ignored when understanding the neural basis and treatment of diseases (Box 1).

Box 1 **A call to action: the use of sex as a factor in research.**

Preclinical studies on depression are essential to our understanding of the disease mechanisms, and in the discovery and screening of novel therapeutics. Despite the higher prevalence of depression in women, and the sex differences in the disease symptomology and pathophysiology, animal models of depression continue to be predominantly carried in male animals, and continue to overlook the role of sex hormones. The “default” use of male animals can be partly attributed to the reluctance of researchers to account for the variability associated with the fluctuation in female hormones (Beery and Zucker, 2011; Zucker and Beery, 2010). Neglecting sex differences and the role of female sex hormones in animal models of depression may be one of the reasons for the poor translation of findings from preclinical to clinical research (Belzung, 2014). The recent move by the National Institute of Health to insist that researchers use both males and females in preclinical studies is a step in the right direction (Clayton and Collins, 2014). The lack of investigation into the influence of sex, however, is not limited to preclinical research. The inclusion of both males and females in clinical trials, as per legislative requirements or recommendations (Merkatz et al., 1993), is not sufficient. There should be a move toward ensuring sufficient male and female sample sizes, and toward analyzing data from clinical trials by sex; i.e., sex should be analyzed as a variable and not merely a covariate to obtain statistically meaningful information on sex differences in depression and antidepressant treatment. Such practices will surely inform our search for new antidepressant treatments that are efficacious and safe in both males and females.

Epidemiological findings consistently show a sex disparity in the lifetime prevalence of depression, with women being twice more likely to be affected (Gutierrez-Lobos et al., 2002). This sex difference in prevalence is seen across cultures (Seedat et al., 2009), emerges during adolescence (Nolen-Hoeksema and Girgus, 1994), and is most apparent during the reproductive years (i.e., 25–50 years; Gutierrez-Lobos et al., 2002). Indeed, there is an increased incidence of depression in women during periods associated with dramatic fluctuations in gonadal hormones particularly during the postpartum and perimenopausal periods (Hendrick et al., 1998; Cohen et al., 2006). Alternatively, when the perinatal period is disturbed, males may be more vulnerable than females to develop other neuropsychiatric diseases, such as autism and schizophrenia, across the lifespan (Stevenson et al., 2000; Kent et al., 2012). Several biological and psychosocial theories have been put forth to explain the underlying cause of the sex differences in depression, but the most prominent neurobiological hypothesis emphasizes the role of gonadal hormones (Hammarstrom et al., 2009). Sex differences in depression extend beyond prevalence rates and course of illness as the symptom profile and clinical presentation differs between men and women. Several studies report that women are more likely to present with comorbid anxiety disorders (Sloan and Kornstein, 2003; Keers and Aitchison, 2010) as well as atypical depression, which is associated with hypersomnia, hyperphagia, or excessive fatigue (Young et al., 1990; Silverstein, 2002). Interestingly, sex is also implicated in antidepressant efficacy, with selective serotonin reuptake inhibitors (SSRIs) being more effective in alleviating symptoms in women, and tricyclic antidepressants (TCAs) being more effective in alleviating symptoms in men (Keers and Aitchison, 2010). However, sex differences in antidepressant efficacy are not always seen (Parker et al., 2003; Dalla et al., 2010). As we discuss later in this review, animal studies of depression have predominantly used male subjects, and thus our understanding of what underlies this differential antidepressant efficacy is limited. The available animal literature on sex-dependent antidepressant efficacy reveals mixed findings (Dalla et al., 2010); for example, some studies suggests that SSRIs are more efficacious in alleviating depressive-like behavior in female rats (Gomez et al., 2014), but others show a higher efficacy in males (Lifschytz et al., 2006). However, the latter study did not account for estrous cycle phase, which may affect depressive-like behavior and antidepressant efficacy. Together these data point to a critical role of sex and gonadal hormones on depression risk, manifestation, and treatment.

#### Stress and HPA involvement in depression

Exposure to chronic stress is tightly linked to the development of depression (reviewed in Tennant, 2002). The hypothalamic-pituitary-adrenal (HPA) axis, a major neuroendocrine stress system (reviewed in Ulrich-Lai and Herman, 2009) exhibits a number of changes in at least a subpopulation of depressed patients, with key features being elevated basal cortisol levels, disrupted diurnal cortisol secretion patterns, and HPA negative feedback dysregulation (Parker et al., 2003; Ising et al., 2007; Schule, 2007; Stetler and Miller, 2011). The HPA negative feedback system can be tested with the administration of dexamethasone, a synthetic glucocorticoid that suppresses cortisol secretion in healthy but not depressed individuals (Carroll et al., 1968; Ising et al., 2007). Chronic treatment with antidepressants can restore the negative feedback function of the HPA axis that either slightly precedes or is coincident with the alleviation of depressive symptoms (Ising et al., 2007). Interestingly, antidepressant effects to normalize HPA negative feedback dysregulation are more tied to remission in women than in men (Binder et al., 2009). Many animal models of depression emphasize the role of stress (reviewed in Yan et al., 2010), and HPA axis dysregulation is used as a measure of a depressive-like endophenotype in such models (Christiansen et al., 2012). Different types of stressors can profoundly influence the effects on depressive phenotypes with generally unpredictable psychological stressors more likely to promote depressive-like behaviors than predictable stressors, which can sometimes provide resilience to depressive-like behaviors (reviewed in McEwen, 2000, 2002; Parihar et al., 2011; Suo et al., 2013). Furthermore, greater allostatic load is associated with more profound effects on depression and the hippocampus (reviewed in McEwen, 2000, 2002). More profound HPA axis dysregulation as a result of chronic stress is seen in female rats when compared to males, marked by larger elevations in corticosterone (CORT), the main glucocorticoid in rodents (Dalla et al., 2005). These findings indicate that HPA dysregulation is seen in both humans and rodents, and this effect may be more profound in females. Moreover, females have naturally higher levels of CORT than males (reviewed in Viau, 2002) and this may contribute to higher incidence of depression in females. It should be noted that other stress hormones such as corticotropin releasing hormone and adrenocorticotropic hormone have been implicated in depression but are beyond the scope of this review and are reviewed elsewhere (e.g., Valentino et al., 2012).

#### The hippocampus and depression

The hippocampus is a highly plastic structure that is sensitive to the effects of stress and sex hormones, both of which are closely linked to depression. A meta-analysis confirmed that untreated depression is associated with a smaller volume of the hippocampus in depressed patients that have been depressed for at least 2 years (McKinnon et al., 2009). The smaller hippocampus associated with depression is an effect that is more prominent in men than women (Frodl et al., 2002) and in middle-aged and older patients (McKinnon et al., 2009). Furthermore, chronic antidepressant exposure appears to increase hippocampal volume in treatment-responding women more so than men (Vakili et al., 2000; reviewed in Lorenzetti et al., 2009). The hippocampus is a highly plastic structure in adulthood (reviewed in Leuner and Gould, 2010), and hippocampal volume fluctuations may be due to changes in neurogenesis, neuropil, and/or apoptosis. Post-mortem studies reveal decreased cell proliferation in the dentate gyrus of the hippocampus of depressed patients (Boldrini et al., 2012). Similarly, hippocampal neurogenesis is reduced in every animal model of depression examined so far (Jaako-Movits et al., 2006; Green and Galea, 2008; Bessa et al., 2009; Brummelte and Galea, 2010a). Chronic but not acute antidepressant treatment restores the depression-model induced decrease in neurogenesis (Green and Galea, 2008; Bessa et al., 2009). Intriguingly, depressed women taking antidepressants have a larger ratio of immature to mature neurons in the hippocampus (an increase in neurogenesis) compared to controls, but the same relationship was not seen in men (Epp et al., 2013). These findings are consistent with the findings that women taking antidepressants show increased hippocampal volume compared to men (Vakili et al., 2000). Furthermore, the effect of antidepressants to induce neurogenesis in the hippocampus is not seen in older depressed patients (Lucassen et al., 2010; Epp et al., 2013), which may be consistent with the lack of efficacy of antidepressants to alleviate depression in older patients (Lenze et al., 2008). Neuropsychological evidence also suggests functional hippocampal impairment in depression, further supporting the role of the hippocampus in the pathophysiology of the disease. For example, a meta-analysis of 726 patients with depression showed neurocognitive impairment, most severely in episodic, declarative memory (Zakzanis et al., 1998), a hippocampal-dependent memory system.

Animal models also show sex-dependent alterations in hippocampal plasticity as a result of chronic stress or chronic corticosterone treatment. Interestingly, chronic footshock stress reduced newly produced cells in the dentate gyrus of individually-housed young adult male rats, but increased new cells in young adult female rats (Westenbroek et al., 2004). Chronic restraint stress reduces neuropil (branch points and dendritic length) in the apical dendrites of CA3 pyramidal cells of the hippocampus in male rats but in the basal dendrites of CA3 pyramidal cells in female rats (Galea et al., 1997). Chronic CORT treatment reduced the density of immature neurons in the dorsal and ventral hippocampus of young adult male rats, but only the ventral hippocampus in young adult female rats (Brummelte and Galea, 2010b). While some researchers suggest that females are less susceptible to the damaging effects of chronic stress than males, this is very much dependent on the nature, duration of the stressor and the background hormonal environment of the female, with generally higher levels of ovarian hormones contributing to fewer damaging effects of stress (Shors et al., 2001; Conrad et al., 2012). Nonetheless these findings suggest that more research is needed to examine how stress alters plasticity in the hippocampus, how these changes in neuroplasticity translate into behavior and disease susceptibility, and how ovarian hormones contribute to this process. Further, stress and ovarian hormones can certainly influence risk for depression and plasticity of other limbic areas, such as the prefrontal cortex, and neurotransmitters, such as serotonin and dopamine, but this is beyond the scope of this review and are addressed elsewhere (e.g., Valentino et al., 2012; Goldstein et al., 2014).

#### Examining depressive-like endophenotypes in animal models

Anhedonia, i.e., the loss of pleasure or interest in previously pleasurable experiences, is one of the core symptoms of clinical depression. Not surprisingly, many animal models of depression focus on modeling anhedonia as a central behavioral endophenotype, typically by measuring the consumption of or preference for sucrose solutions. While reductions in the hedonic value of sucrose as a result of chronic unpredictable stress is seen in both male and female rats, the effect is more profound in male rats (Grippo et al., 2005; Dalla et al., 2005, 2008; Kamper et al., 2009). Because sucrose consumption/preference tests were first developed in male rodents, and because baseline sucrose consumption in females may fluctuate with the estrous cycle (Clarke and Ossenkopp, 1998), it may not be an ideal model of anhedonia in female rodents. On the other hand, other stress-induced depressive-like behaviors are more evident in female rats. For example, female rats show more despair-like behavior (immobility) on the forced swim test following chronic mild stress (Sachs et al., 2014) but not following chronic CORT treatment (Kalynchuk et al., 2004), an effect that may be related to sex differences in basal CORT levels (reviewed in Viau, 2002). As mentioned earlier, there are also reported sex differences in the symptoms of depression and in the prevalence of comorbid disorders; women are more likely to present with co-morbid anxiety and somatic complaints, whereas men are more likely to present with co-morbid alcohol and substance abuse (Marcus et al., 2008). Clearly, more clinical and preclinical research is needed to examine sex differences in the symptoms, and in the treatment alleviation of certain symptoms of depression.

The current article explores how adverse events present during developmental windows such as the prenatal period, the early postnatal period, and adolescence, are linked to increased vulnerability to neuropsychiatric disorders in adulthood. Many sex differences in the brain are programmed early in life (Paus, 2010). It perhaps is not surprising then that the sex differences associated with neuropsychiatric disorders also have a developmental component. The following sections will outline how prenatal, postnatal, adolescent or adult perturbations in stress or excessive stress hormones influence vulnerability to develop depression in both humans and animal models with a special emphasis on the behavioral analysis, HPA axis modulation, and neuroplasticity in the hippocampus.

### Prenatal manipulations and vulnerability to depression

Prenatal stress limits fetal growth and gestational length (reviewed in Seckl and Holmes, 2007), and is consequently associated with an increased risk for depression, anxiety, schizophrenia, and more recently autism (reviewed in Bale et al., 2010; Baron-Cohen et al., 2014). During gestation, the placental enzyme 11β-hydroxysteroid dehydrogenase type II inactivates excessive levels of maternal or exogenous cortisol to protect the fetus from high cortisol. However, there are sex differences in the response of this placental enzyme to stress exposure which could contribute to which sex is more vulnerable to excessive maternal stress (reviewed in Clifton, 2010). For example, the female, but not male, placenta exhibits an increase in 11β-hydroxysteroid dehydrogenase type II expression in response to prenatal exposure to betamethasone (a synthetic glucocorticoid) prior to preterm delivery (Stark et al., 2009). Although seemingly contradictory to the general pattern that females are more vulnerable to depression than males, this finding is consistent with findings that males are more vulnerable to neurodevelopmental disorders that can be triggered during gestation, such as autism, and poor outcome after preterm birth (Stevenson et al., 2000; Kent et al., 2012). Thus, the developmental timing of stress exposure may play a pivotal role in which sex is more likely to develop depression and the reader is directed to excellent reviews on the subject (Andersen, 2003; Teicher et al., 2003; Brenhouse and Andersen, 2011). Further research investigating how sex differences in placental function mediate developmental outcome will provide a better understanding of why males are more vulnerable to the effects of prenatal stress than females.

#### Prenatal stress affects depressive behavior

Gestational stress can contribute to the development of mood disorders in the mother during pregnancy (Lancaster et al., 2010). Depression during pregnancy in the mother increased emotional responses in infant boys, but not girls (Gerardin et al., 2011), and increased risk for depression in adolescence (Pawlby et al., 2009; Pearson et al., 2013). Furthermore, maternal anxiety during pregnancy is associated with a greater risk for depressive symptoms in adolescent girls (Van Den Bergh et al., 2008) and a greater risk for attention deficits in young and adolescent boys (Van Den Bergh et al., 2006; Loomans et al., 2011). Together, these studies suggest that maternal mood during gestation may differentially affect behavioral outcome in boys and girls.

Animal models of depression that induce prenatal stress have been used to examine how early life perturbations differentially affect male and female offspring. Generally these animal models involve exposing the pregnant dam to predictable or unpredictable stress (reviewed in Weinstock, 2008). Despite the fact that clinical research points to strong associations between prenatal stress and likelihood to develop depression, preclinical research has yielded mixed results. However, it should be noted that the third trimester equivalent in rodents is the first week postnatal and thus one reason why there may be differences between human and animal studies is timing of “trimester” in different species (Kleiber et al., 2014). Timing of stress onset plays a critical role in how it affects offspring depressive-like behavior. For example, one study found that prenatal stress increased depressive like behavior (increased immobility in the forced swim test) of adult male mice only when stress occurred during the first week of gestation but not during mid- or late-gestation (Mueller and Bale, 2008) which would be akin to the first vs. second trimester. However, others have shown that prenatal restraint stress during the last week of gestation (akin to late in the second trimester) increased depressive-like behavior (increased immobility in the forced swim test) in adult males and females, although in males it is only seen when restraint occurs three times per day during the last week of gestation (Alonso et al., 1991; Morley-Fletcher et al., 2003; Szymañska et al., 2009; Van Den Hove et al., 2014). Age of offspring at behavior testing is also critical as prenatal stress had the opposite effect on depressive-like behavior (decreased immobility in the forced swim test) in pre-pubertal (P33; Schroeder et al., 2013) and adolescent male and female rats (Rayen et al., 2011). Alternatively, direct administration of CORT to the dam during days 10–20 of gestation (equivalent to second trimester) increased depressive-like behavior (increased immobility in the forced swim test) for both adolescent male and female rats (Brummelte et al., 2012). Ultimately, duration of prenatal stress, frequency of stress exposure, timing of prenatal stress, type of stressor employed, species, and age of offspring at testing influence depressive-like behavior of the offspring (reviewed in Huizink et al., 2004; Weinstock, 2011). Additional research addressing how these differences in methodologies influence offspring outcome will be valuable for understanding how prenatal factors influence vulnerability to depression in males and females.

#### Prenatal stress affects development of HPA axis

Prenatal stress can have variable programming effects on the HPA axis of offspring depending on sex, the paradigm of prenatal stress and the part of the HPA axis (basal, stress peak, stress recovery) analyzed (Barbazanges et al., 1996; Zagron and Weinstock, 2006). For instance, prenatal stress for even 1 week of gestation can result in prolonged CORT recovery after acute stress in adult female but not in adult male rats (Weinstock et al., 1992; McCormick et al., 1995). However, after more intense prenatal stress (stress three times per day during last week of gestation and equivalent to second trimester) even male rats displayed prolonged CORT recovery after restraint (Maccari et al., 1995; Morley-Fletcher et al., 2003) and both sexes exhibit disrupted diurnal CORT rhythm (Koehl et al., 1999). Alternatively, social defeat stress during the last week of gestation (equivalent to second trimester) exaggerated the peak CORT response after restraint in both adult male and female rats (Brunton and Russell, 2010). These findings suggest that more intense paradigms of prenatal stress are capable of reprogramming the male HPA axis whereas females seem to be sensitive to milder forms of prenatal stress.

In clinical studies, prenatal stress has been associated with HPA axis dysregulation in infants, adolescents, and adults (reviewed in Glover et al., 2010). However, there are limited studies directly assessing how sex mediates the effects of prenatal stress on the development of the HPA axis. Both maternal prenatal anxiety and prenatal exposure to synthetic glucocorticoids (either dexamethasone or betamethasone) increased stress reactivity in girls only (De Bruijn et al., 2009; Alexander et al., 2012). However, whether HPA axis dysregulation persists in girls or whether it emerges later on in boys remains unknown. Future clinical studies directly analyzing how sex mediates the relationship between prenatal stress and development of HPA axis are necessary to address this gap.

#### Prenatal stress affects hippocampal plasticity

Prenatal stress persistently decreased cell proliferation in the hippocampus of juvenile, adolescent, adult, and aged male rodents (Lemaire et al., 2000; Mandyam et al., 2008; Rayen et al., 2011; Belnoue et al., 2013) and in pre-pubescent rhesus monkeys (2–2.5 years old; Coe et al., 2003). The prenatal stress-induced decrease in cell proliferation is more prominent in the ventral hippocampus (Zuena et al., 2008) and the ventral hippocampus is associated with stress and anxiety more so than the dorsal hippocampus (reviewed in Fanselow and Dong, 2010). In contrast, the effect of prenatal stress on hippocampal cell proliferation in female offspring is more complicated. Prenatal stress decreased hippocampal cell proliferation in adolescent female rats (Rayen et al., 2011) but had variable effects in adult females. Prenatal stress diminished cell proliferation in adult (5 months old) female rats (Mandyam et al., 2008) and in aged (2 year old) females (Koehl et al., 2009) but the later study did not see the same effect in younger adult female offspring. Although both studies employed the same prenatal stress paradigm, there were differences in onset of prenatal stress (gestational day 14 or 15) as well as breeding (shipped pregnant vs. breeding in house) that may explain this conflict (Laroche et al., 2009). Prenatal stress diminished number of immature neurons (doublecortin expressing) in adolescent male and female rats (Rayen et al., 2011) and decreased neurogenesis in adult male rodents (Lemaire et al., 2000; Belnoue et al., 2013). Prenatal stress affects other aspects of hippocampal morphology as well as other regions of limbic system and are reviewed elsewhere (e.g., Charil et al., 2010; Weinstock, 2011). In non-human primates, there are known effects of prenatal excessive glucocorticoids to show damage to different areas of the hippocampus (reduction in volume) into middle age (Uno et al., 1994). In humans, one study found that prenatal stress diminished use of hippocampal-dependent strategies in a spatial task (Schwabe et al., 2012). However, whether these effects are paralleled in hippocampal volume, related to risk for depression, or differentially affected by sex remain unknown and likely need to be taken into account to fully understand the effects of prenatal and postnatal adversity on the hippocampus in humans (Frodl and O'Keane, 2013).

While there has been robust evidence from preclinical and clinical studies that prenatal stress negatively affects offspring outcome, prenatal stress can also diminish quality of maternal care (Champagne and Meaney, 2006) and even serve as a model of postpartum depression (PPD; Smith et al., 2004; Leuner et al., 2014). Thus, studies that employ prenatal stress paradigms essentially result in a mix of prenatal and postnatal adversity, obscuring the degree to which poor functional outcome can be attributed to *in utero* stress alone. Cross-fostering of prenatally stressed with non-stressed rodent pups reverses certain behavioral and endocrine effects (Barros et al., 2006; Del Cerro et al., 2010; Perez-Laso et al., 2013). In humans, studies have found that some of the negative effects of prenatal stress on child outcome are mediated by the early postnatal environment, such as presence of postpartum mood disorders or poor maternal care (Kaplan et al., 2008; Bergman et al., 2010; Rice et al., 2010). These studies highlight the prenatal and postnatal environments as potent mediators in offspring development.

### Postnatal manipulations and vulnerability to depression

#### Postnatal stress affects depressive behavior

Several forms of postnatal early life stress, such as sexual, physical, or emotional abuse as well as parental loss, neglect, or mental illness constitute increased risk for adult mood and anxiety disorders (Famularo et al., 1992; Pelcovitz et al., 1994; reviewed in Heim and Nemeroff, 2001). In fact, recent work suggests that depression is harder to remit if the individual has a history of early childhood adversity such as physical abuse (Fuller-Thomson et al., 2014). One of the most potent elements of early life stress is the quality of parental care (Ladd et al., 2000). Indeed, postpartum mood disorders such as PPD disrupt a healthy mother–infant bond and can have a profound effect on child development (reviewed in Goodman and Gotlib, 1999; Lovejoy et al., 2000; Ashman et al., 2002; Deave et al., 2008; Van Hasselt et al., 2012). For example, boys of PPD mothers have lower IQ (reviewed in Grace et al., 2003; Azak, 2012) while both adolescent girls and boys of PPD mothers are more likely to develop depression and anxiety (Pilowsky et al., 2006; Murray et al., 2011) as well as a propensity for violent behavior (Hay et al., 2003). Of course PPD is not often seen in isolation, and antenatal depression (as described above) or maternal depression can also influence emotional behaviors such as antisocial behavior in children (Kim-Cohen et al., 2005; Hay et al., 2010). Indeed maternal depression, which is often defined as depression within a year after giving birth, can critically affect cognitive and emotional development of children (reviewed in Goodman and Gotlib, 1999). The reader is directed to a review on maternal depression during gestation or postpartum differentially affecting child outcome (Hay et al., 2008). Thus, PPD alone or in conjunction with additional depressive episodes can profoundly influence child development and represents a risk for behavioral and cognitive disturbances.

Disruptions to the early postnatal environment have also been linked to depressive-like behavior in animal models of depression. One of the most common postnatal paradigms used is maternal separation in which pups are removed from the dam for 3 hours per day during the postnatal period (reviewed in Schmidt et al., 2011). As may be expected, offspring outcome after maternal separation differs in terms of the duration, timing and method of separation (dam or pups removed from home cage). Again it is worth noting that the first week of postnatal life in rodents is equivalent to the third trimester in humans. Maternal separation during the first two postnatal weeks (equivalent to third trimester) or the entire postnatal period (from birth to weaning) increased immobility in the forced swim test in adult male and female rats (Lee et al., 2007; Aisa et al., 2008; Lajud et al., 2012). Another interesting model of postnatal adversity is the limited bedding model in which dams are provided with limited bedding and nesting material during the early postnatal period (equivalent to third trimester). Denying the mother sufficient nesting material consequently decreased maternal behaviors and increased erratic and abusive maternal behaviors (Ivy et al., 2008) which result in increased immobility in the forced swim test in adolescent rat offspring (Raineki et al., 2012). Thus, substantial loss of proper maternal care during the postnatal period can impact both male and female vulnerability to depression and sensitivity to stress well beyond the time they are dependent on the mother.

#### Postnatal stress affects development of HPA axis

Maternal separation influences HPA axis development in a time and sex-specific manner. For instance, maternal separation during the first two postnatal weeks (equivalent to third trimester) reduced basal CORT in adult females but not in adult males (Slotten et al., 2006). However, maternal separation lasting the entire postnatal period elevated basal CORT in adult females (Aisa et al., 2008). Maternal separation during the first 2 weeks or entire postnatal period blunted the peak CORT response to stress in pre-weaning (Litvin et al., 2010) and adolescent male rats (Ogawa et al., 1994). However, by adulthood, maternal separation had no significant effect on CORT reactivity in either adult male rats or female rats (Plotsky and Meaney, 1993; Plotsky et al., 2005; Slotten et al., 2006). Interestingly, maternal separation during the first two postnatal weeks (equivalent to third trimester) resulted in exaggerated adrenocorticotropic hormone secretion after restraint (Liu et al., 2000) but not after exogenous corticotropin releasing hormone in male rats (Wigger and Neumann, 1999), suggesting that maternal separation may have a more potent effect on sensitivity of the pituitary gland to different stressors in male rats. Maternal separation in rodents closely approximates parental neglect in humans, which is also associated with disruptions to the developing HPA axis (reviewed in Tarullo and Gunnar, 2006). For example, toddlers raised in the extreme psychosocial neglect in Romanian orphanages exhibit blunted secretion of cortisol over the day (Carlson and Earls, 1997) but exhibit exaggerated cortisol secretion as young children (Gunnar et al., 2001). Similarly, PPD and early life abuse are associated with HPA axis hyperactivity in adolescents and adult men and women, respectively (PPD: Halligan et al., 2004; abuse: Heim et al., 2002, 2008). However, timing of abuse onset is a critical factor as if the onset of abuse was early, then young girls present with hypocortisolemia whereas young boys present with hypercortsolemia (Doom et al., 2013). Together, these data suggest that the postnatal environment can either blunt or exaggerate HPA axis activity depending on sex, timing and type of postnatal stress.

#### Postnatal stress affects hippocampal plasticity

Maternal separation lasting the first two postnatal weeks (equivalent to third trimester) diminished cell proliferation, but not the survival of new neurons in the dentate gyrus, in adult male rats, which was reversed by adrenalectomy (Mirescu et al., 2004). Maternal deprivation for 24 h on postnatal day 3 enhanced number of immature neurons in male rats but diminished number of immature neurons in female rats at weaning (Oomen et al., 2009). However, by adulthood, the number of immature neurons and survival of new neurons was diminished in the ventral hippocampus of adult male rats but not in female rats (Oomen et al., 2010, 2011). This suggests that males experience more dynamic and long-lasting changes to neurogenesis than females in response to maternal separation. In humans, children exposed to maternal depression since birth exhibit no significant change in hippocampal volume (Lupien et al., 2011) whereas adult hippocampal volume is vulnerable to the effects of childhood maltreatment (Chaney et al., 2014). This may be partly explained by low socioeconomic status which is associated with reductions in hippocampal volume in children/adolescents (Noble et al., 2012) as well as increased risk for PPD (Goyal et al., 2010). There are also sex differences as another study found that self-reported low maternal bonding was associated with small hippocampal volume in women but not in men (Buss et al., 2007). Future research addressing how maternal care interacts with socioeconomic status to affect hippocampal volume will help shed light on how the hippocampus can be affected by maternal care and the postnatal environment throughout the lifespan.

While maternal separation has provided clear evidence that separation from the dam alters offspring development, there are caveats to this model. For instance, there are changes in maternal care that occur based on the separation and reuniting of the dam with her pups (Pryce et al., 2001; Macrí et al., 2004; Own and Patel, 2013). Moreover, maternal separation results in increased depressive like behavior in the dam (increased immobility in the forced swim test; Boccia et al., 2007). Our laboratory has developed a model of PPD in which dams are treated with high levels CORT (40 mg/kg/day) throughout the postpartum period which diminished maternal care and increased immobility in the forced swim test in the dam (Brummelte et al., 2006; Brummelte and Galea, 2010a). Because our model requires an injection to the dam, there is minimal separation (less than 1 min of separation each day) of the pups from their mother. Thus, our model more closely resembles the consistent levels of voluntary diminished maternal care or neglect typical of PPD as opposed to forced sessions of total maternal deprivation. We have shown that high maternal postpartum CORT increased anxiety-like behavior in adolescent male, but not female, rats (Brummelte et al., 2012). Furthermore, high maternal postpartum CORT diminished cell proliferation in the dentate gyrus of juvenile male but not female rats (Brummelte et al., 2006). CORT during pregnancy and the postpartum also increased peak CORT in response to restraint stress in both males and females (Brummelte et al., 2012). Altogether, these data suggest that high maternal CORT affects offspring HPA axis, hippocampal plasticity and anxiety-like behavior in a sex-specific manner, consistent with the effects of PPD on child development.

### Adolescence manipulations and vulnerability to depression

#### Adolescent stress affects depressive behavior

While the perinatal period has received much attention in terms of predisposing individuals to mood disorders, recent research has highlighted the importance of the adolescent period as schizophrenia, substance abuse, depression, and anxiety can emerge during adolescence (Costello et al., 2003; reviewed in Patton and Viner, 2007). It should be noted that adolescence broadly refers to the developmental window involving the transition from childhood to adulthood whereas puberty specifically refers to the maturation of the HPG axis and the subsequent gonadal hormone fluctuations occurring during adolescence (Spear, 2000). Stressful events during adolescence might have compounding effects on an already volatile state of emotional and social challenges during adolescence (Spear, 2000). Thus, stress exposure during the adolescent years could influence risk for depression as well as an increased vulnerability to neuropsychiatric disorders later in life (reviewed in McCormick and Mathews, 2007; Holder and Blaustein, 2014). Further, as indicated above, the consequences of perinatal stress may emerge during adolescence or even interact with adolescent stress to shape vulnerability to adult depression (Rueter et al., 1999; Goodyer, 2002). For instance, the presence of both antenatal depression and child maltreatment increased risk of depression by almost five-fold and increased risk of developing psychopathologies by twelve-fold in adolescents (Pawlby et al., 2011). Alternatively, positive family support can reduce the risk for depression in adolescent boys and girls depending on genetic background, supporting the idea that postnatal factors mediate risk for adolescent depression (Li et al., 2013).

The rise in gonadal hormones during puberty plays an important role in precipitating the sex differences in depression. In fact, as mentioned earlier the increased female prevalence of depression emerges during puberty (Ge et al., 1996). Interestingly, adolescent female rats spend more time immobile in the forced swim test than adolescent male rats even without any additional experimental manipulations (Leussis and Andersen, 2008). This suggests that animal models are also able to capture this early female vulnerability to depression. Stressors during adolescence can also differentially affect adolescent male and female depression-like behavior. Social isolation during early adolescence (P30-35) increased depressive-like behaviors (immobility in the forced swim test and increased latency to escape shock in the shuttlebox) in adolescent male, but not female, rats (Leussis and Andersen, 2008). Similarly, social defeat stress during early adolescence (P29-31; P35-44, respectively) increased immobility in the forced swim test in adolescent male, but not female, rodents (Ver Hoeve et al., 2013; Iñiguez et al., 2014). However, when early adolescent rats are exposed to alternating episodes of social defeat and restraint, adolescent female, but not male, rats exhibited shorter latency to immobility in the forced swim test which persisted into adulthood (Bourke and Neigh, 2011). This suggests that perhaps adolescent males are more sensitive to social stressors but adolescent females are more sensitive to multimodal stressors. Others have found an opposite effect of adolescent social isolation only in females such as increased climbing behavior in the forced swim test (but not immobility) in adolescence and adulthood as well as increased sucrose preference in adulthood (Hong et al., 2012). Given that adolescence is characterized by a complex array of social behaviors and challenges, it is possible that there are sex differences in how adolescent animals are vulnerable to the effects of social stress. This may be an important point to consider in animal models assessing adolescent vulnerability to depression.

#### Adolescent stress affects HPA axis

The responsiveness of the HPA axis to stress during adolescence is different than that of the adult HPA axis (reviewed in Klein and Romeo, 2013). Additionally, the maturation of the HPA axis is dependent on the maturation of the HPG axis (Romeo, 2010) and the increased risk for depression during adolescence may be related to maturation of the HPA-HPG interactions (reviewed in Angold and Costello, 2006). After 5 days of restraint stress, CORT levels habituate in adolescent female, but not male, rats (Doremus-Fitzwater et al., 2009). Chronic social instability stress during adolescence exaggerates the peak CORT response after swim stress in adolescent male and female rats, but this effect is not seen in adulthood (Mathews et al., 2008). Chronic restraint stress throughout adolescence (P30-P52) significantly increased basal CORT levels for adult females, but not male, rats (Barha et al., 2011). However, 1 week of restraint during early adolescence (P26-33) blunted HPA axis reactivity in adult male rats but had no significant effect on adult female rats (Ariza Traslavina et al., 2014). Thus, hyperactivity of the HPA axis is observed in both males and females during adolescence, but timing of adolescent stress exposure results in sex differences in terms of the longitudinal effects on adult HPA axis. Early adolescent stress causes HPA hypoactivity in adult males (Ariza Traslavina et al., 2014) but stress throughout adolescence causes HPA hyperactivity in adult females (Barha et al., 2011).

#### Adolescent stress affects hippocampal plasticity

Chronic restraint stress throughout adolescence diminished neurogenesis (cell proliferation and survival) in the dentate gyrus in adult female rats, but slightly increased neurogenesis in adult male rats (Barha et al., 2011). Interestingly, this same pattern persists in social stress paradigms as social instability stress during adolescence diminished hippocampal cell proliferation in adolescent female rats (McCormick et al., 2010) while it enhanced cell proliferation and doublecortin-expressing cells in the dorsal hippocampus of adolescent male rats (McCormick et al., 2012). Moreover, social isolation during adolescence also diminishes cell proliferation specifically in the rostral hippocampus and immature neurons throughout the hippocampus in adolescent marmoset monkeys (Cinini et al., 2014). Unfortunately although while both male and female marmosets were used in the study, sex was not independently analyzed, so it is unclear whether sex differences were present in their findings to match those of previous studies in rodents (Cinini et al., 2014). Furthermore, chronic resident-intruder stress did not significantly affect hippocampal cell proliferation in adolescent male rats (Hanson et al., 2011). Together, these studies highlight the importance of stress and the adolescent period on hippocampal neurogenesis and that females are more likely to show reduced neurogenesis in response to adolescent stress compared to males. Both preclinical and clinical research has been investigating how stressors occurring during adolescence affect behavior and maturation of the brain. Future studies should also examine effects of adolescent stress on hippocampal volume and function both during adolescence and later in adulthood to help bridge the gap preclinical research with clinical research (reviewed in Andersen and Teicher, 2008; McCormick and Green, 2013).

### Adult manipulations and vulnerability to depression

#### Depression in the postpartum

As discussed previously, PPD and other maternal mood disorders can affect child's risk for depression. Interestingly, the postpartum period is the time of greatest risk for women to develop depression (Drevets and Todd, 2005) and affects approximately 15% of women (Kornstein, 2002; Goodman, 2007; Wisner et al., 2013). To complicate matters, women are reluctant to take antidepressants during pregnancy or the postpartum with only 18% of depressed mothers seeking treatment (Marcus, 2009). During pregnancy and postpartum, levels of steroid hormones fluctuate dramatically which could contribute to the etiology of PPD (Hendrick et al., 1998; Bloch et al., 2003). Pregnancy and postpartum are characterized by sustained high flattened levels of glucocorticoids in both humans (Magiakou et al., 1996; Schule, 2007) and rodents (Lightman et al., 2001; Pawluski et al., 2009), which is a similar hormone profile observed in depressed patients (Stetler and Miller, 2011). Women who previously suffered from PPD reported more depressive symptoms and showed greater cortisol responses after exposure to a hormone-simulated pregnancy (Bloch et al., 2005), suggesting that in vulnerable women, the HPA axis and mood are altered in response to pregnancy hormones. An animal model of depression from our laboratory has shown that high levels of CORT directly administered to the dam increased depressive-like behavior (increased immobility in the forced swim test) and decreased dendritic complexity and cell proliferation in the hippocampus at the end of the postpartum period (Brummelte et al., 2006; Brummelte and Galea, 2010a; Workman et al., 2013). The ability of chronic antidepressants to increase neurogenesis in the hippocampus is tied to the liable nature of corticosterone (Huang and Herbert, 2006). Given the increased and flattened profile of cortisol during pregnancy and postpartum, it may not be surprising then that antidepressants prescribed at this time may not be as efficacious as they would be in cycling women. Indeed, there is little evidence to suggest that prescribed antidepressants work better than psychotherapy or placebo methods during the postpartum (reviewed in O'Hara and McCabe, 2013; De Crescenzo et al., 2014). In addition to changes in HPA hormones across pregnancy and the postpartum, estradiol levels are elevated throughout the third trimester but drop dramatically after parturition, leading to the hypothesis that an “estradiol-withdrawal state” during the first few weeks after parturition contributes to PPD (Hendrick et al., 1998; Bloch et al., 2003). Our laboratory was first to show that withdrawal from a hormone-simulated pregnancy induced depressive-like symptomology (increased immobility in the forced swim test and sucrose anhedonia; Galea et al., 2001; Green et al., 2009) and reduced adult hippocampal neurogenesis in female rats (Green and Galea, 2008). There is also evidence that the postpartum period is associated with reduced plasticity in the hippocampus (Pawluski and Galea, 2006, 2007) and neuroplasticity is thought to be integral to antidepressant efficacy, as further discussed below (Wainwright and Galea, 2013).

#### Depression in perimenopause

The perimenopausal period is characterized by dramatic fluctuations in hormones followed by a persistent hypogonadal state (Burger et al., 2008). The period of transition into menopause lasts approximately 10 years and poses an increased risk to develop depression for women (Freeman et al., 2004; Cohen et al., 2006), suggesting a role of gonadal hormones in the etiology of the disease. Although a previous history of depression is a strong predictor of depression in the perimenopausal period (Freeman et al., 2004), the risk is also increased in women with no previous history of the disease (Cohen et al., 2006; Freeman et al., 2006). Antidepressant efficacy is also associated with the gonadal hormone status of postmenopausal women. In postmenopausal women with depression, SSRIs show better efficacy in those on hormone therapy (HT) in comparison with those not receiving HT (Thase et al., 2005; Pae et al., 2009; Kornstein et al., 2010), suggesting the efficacy of HT as an adjunct therapy alongside SSRI antidepressants in postmenopausal women. Similarly, the gonadal hormone status of men is implicated in depression, and correlational studies indicate that depressed men have lower levels of testosterone (Sachar et al., 1973; McIntyre et al., 2006). Interestingly, this effect seems to be more prominent in aging men, in which the age-related decline in testosterone may be related to depression (Carnahan and Perry, 2004). Moreover, testosterone as an adjunct therapy with antidepressants shows efficacy in alleviating depression symptoms in hypogonadal men (Seidman et al., 2001, 2009), a finding that was also supported by a meta-analysis (Zarrouf et al., 2009). Thus, the decline in gonadal hormones in aged men and women may predispose vulnerable individuals to depression and HT may be efficacious as an antidepressant adjunct therapy.

#### Depression in older age

With the aging of populations and the increasing life expectancy, interest in older-age depression has increased. Older-age depression often presents with comorbid medical conditions (Alexopoulos et al., 2002), is associated with poorer outcomes of comorbid illnesses (Sinyor et al., 1986; Palinkas et al., 1990; Michelson et al., 1996; Musselman et al., 1998) and higher rates of mortality (Rovner et al., 1991; Ganguli et al., 2002). Methodological differences may account for the wide range of reported prevalence rates of major depression in older individuals, which can be up to 9.4% in the community and 42% in nursing homes (reviewed in Djernes, 2006). In close to half of older individuals with depression, the illness takes on a chronic or relapsing course (Weyerer et al., 1995; Mojtabai and Olfson, 2004). Furthermore, the efficacy of antidepressants in this population is poor; this is evident in the high rates of treatment resistance in randomized control trials using first-line antidepressants, which are up to 81% with serotonin/norepinephrine reuptake inhibitors, and up to 77% with SSRIs (reviewed in Lenze et al., 2008). Treating older-age depression is further complicated by factors related to the higher comorbidity of medical illnesses, such as interactions with drugs frequently prescribed to older individuals (Spina and Scordo, 2002). Unfortunately, older-age depression is also under-diagnosed and/or under-treated (Steffens et al., 2000; Watson et al., 2003; Stek et al., 2004); this may be in part due to a unique symptom presentation in which older individual with depression are more likely to report somatic complaints rather than depressed mood (Small, 1991). Unfortunately there is very little information on whether sex differences are still seen in this population.

Major depression often presents in the context of dementia, with prevalence rates of about 17% in patients with Alzheimer's disease (Wragg and Jeste, 1989). Depression in the older population accelerates dementia and is associated with poorer cognitive outcomes (Bromberger et al., 2003). Interestingly, older individuals with mild cognitive impairment are more likely to develop dementia after diagnoses with major depression (Freeman et al., 2004; Schmidt et al., 2004). Moreover, evidence regarding the efficacy of antidepressant treatments in patients with dementia is weak; in a meta-analysis where only four randomized control trials met inclusion criteria, the authors suggest that there is no sufficient evidence to conclude that antidepressants are efficacious in individuals with depression and dementia (Bains et al., 2002). More recent meta-analyses also showed that neither response nor remission rates differ between antidepressant treatments and placebo in this population (Nelson and Devanand, 2011; Sepehry et al., 2012). With the continued growth of the older population, older-age depression should be regarded as a serious public health concern, and efforts to enhance its recognition and treatment are essential.

#### HPA function in older-age and its potential contribution to depression

Interestingly, there are marked changes in HPA function related to older-age. Cortisol levels increase dramatically (Van Cauter et al., 1996), and the normal fluctuations in diurnal cortisol rhythms are blunted with age (Ferrari et al., 2001). Furthermore, a meta-analysis showed that older-age is associated with HPA negative feedback dysregulation, which is significantly more prominent in women (Otte et al., 2005). Intriguingly, and as described earlier, such changes in HPA function mirror those seen in depressed patients regardless of age, and may therefore contribute to the increased risk of depression in older individuals. However, how sex differences in HPA function in this population may contribute to differences in depression risk or presentation is unclear and warrants further research.

#### Neuroplasticity in older-age may be implicated in antidepressant efficacy

While the neurobiology behind the lowered antidepressant efficacy in older individuals is not well understood, it may be explained by aging-related changes in neuroplasticity, particularly in the hippocampus. As mentioned above, a meta-analysis on hippocampal volume and depression found that a smaller volume of the hippocampus was associated with depression particularly in the older population (McKinnon et al., 2009). Further, many aspects of hippocampal plasticity are reduced in older-age, for example, there is a decline with age in neurogenesis levels (Couillard-Despres, 2013), synaptic proteins (Eastwood et al., 1994; VanGuilder et al., 2010), and spine densities (Anderson and Rutledge, 1996; Von Bohlen Und Halbach et al., 2006; Tsamis et al., 2010) in humans as well as rodents. Additionally, older-age is associated with reductions in serum brain-derived neurotrophic factor (Ziegenhorn et al., 2007), which is important for neural plasticity and compromised in depression (reviewed in Autry and Monteggia, 2012). The age-related reductions in gonadal hormones in both men (Ferrini and Barrett-Connor, 1998) and women (Burger et al., 2008) are, at least in part, responsible for the lowered malleability of the brain. Indeed, gonadal hormones are known to regulate neuroplasticity on many levels (Yang et al., 2004; Franklin and Perrot-Sinal, 2006; reviewed in Galea et al., 2013; Hamson et al., 2013). This reduced state of plasticity may render the brains of older individuals less malleable in response to antidepressant treatments. In fact, antidepressant-induced increase in neurogenesis is not seen in older depressed patients (Lucassen et al., 2010; Epp et al., 2013). This association between the lower state of plasticity and lower antidepressant efficacy aligns with newer theories of depression suggesting that alterations in neuroplasticity, beyond neurogenesis, may serve as a unifying mechanism through which antidepressant drugs produce their effect (reviewed in Wainwright and Galea, 2013). Therefore, if antidepressants work through neural remodeling, then antidepressant failure may result from an impaired ability of the nervous system to undergo change (“plasticity potential”). Evidently, preclinical and clinical research is warranted to explore this possibility.

#### Neuroplasticity and antidepressant efficacy during the postpartum

Curiously, low plasticity potential may also explain vulnerability to depression and/or reduced antidepressant efficacy in other populations, for example, women in the postpartum period. There is a shortage of randomized controlled trials investigating the effectiveness of antidepressants in the postpartum, however, the current evidence does not suggest that commonly prescribed antidepressant treatments are efficacious in this population (reviewed in O'Hara and McCabe, 2013; Sharma and Sommerdyk, 2013). Magnetic resonance imaging studies show a decrease in brain size and an increase in ventricular size during pregnancy, which persist in the postpartum period until 6 months post-delivery (Oatridge et al., 2002). The changes that occur in the maternal brain are poorly understood, and the mechanisms behind this decrease in size cannot be explained using imaging studies. Animal research supports the theory that early postpartum is associated with reduced plasticity at least in the hippocampus (Pawluski and Galea, 2006, 2007; Darnaudéry et al., 2007; Leuner et al., 2007; reviewed in Hillerer et al., 2014). Clearly, there are large changes in neuroplasticity associated with pregnancy and the postpartum, and a lower plasticity state may explain the reduced antidepressant efficacy by traditional antidepressants in this group. Thus, not only do we need to better understand how depression is different between women and men, but also how different periods within a woman's life can impact the development and treatment of depression.

## Conclusions

The evidence we review here, with a focus on depression, suggests that sex interacts with developmental window and age to modulate the outcomes of stress exposure. Several facets of depression are differentially impacted by the interaction of sex and age, including vulnerability, symptomology, treatment efficacy and pathophysiology, as indicated by differences in HPA function and hippocampal plasticity. We highlight that males and females are differentially vulnerable to different types of stress across the lifespan, with males being more vulnerable to perinatal perturbations, females being more vulnerable later in life during peripartum, and both sexes being affected in adolescence and aging. These findings are summarized in Tables 1–3. Thus, in order to best understand depression and improve its treatment, researchers must not only utilize both sexes, but should acknowledge that evaluation and treatment in one sex may not be optimal for the other sex (for further discussion, please see Box 1). Moreover, given that pregnancy and the postpartum are accompanied by dramatic physiological changes, it should not be surprising that depression and antidepressant efficacy are different in these women compared to cycling women. Finally, we highlight the need for an improved understanding of depression and its treatment in older-age. We provide evidence suggesting that the lowered state of neuroplasticity in the older brain may contribute to the disease etiology and/or lowered antidepressant efficacy in this population. To conclude, we advocate for the inclusion of more female subjects, and for the analysis of results by sex in both preclinical and clinical research, for it is vitally important to begin to do systematically, as only in this way will we start to understand the powerful effects of sex on stress and depression risk.

**Table 1:** **Effects of stress exposure at different time points throughout life on depressive-like behavior (i.e., immobility in the forced swim test; FST) in preclinical research (first row) and on depression in clinical research (second row)**.

**Vulnerability**	**Prenatal**	**Postnatal**	**Adolescent**	**Adult**
♂ > ♀	↑ Immobility in FST • Variable stress, GD 1–7 (173) • Restraint 3×/day, GD 14–21 (172, 241, 254)	–	↑ Immobility in FST • Isolation (in adolescence; 141) • Social defeat (in adolescence, 115)	↓ Sucrose preference • Chronic mild stress (57, 120) ↑ Immobility in FST • Chronic corticosterone administration (119)
	–	–	–	–
♀ > ♂	↑ Immobility in FST • Restraint and suspension, GD 15–21 (254)	–	↑ Immobility in FST • No stress (in adolescence; 141) • Social defeat and restraint (in adolescence, 29)	↑ Immobility in FST • Chronic mild stress (213)
	↑ Depression • Antepartum anxiety (in adolescence, 253)	–	↑ Depression • No stress (84)	↑ Depression • No stress (98) • Perimenopause and postpartum (51, 109) ↑ Atypical depression • No stress (226, 273) ↑ Comorbid disorders • No stress (122, 228)
♀ = ♂	↑ Immobility in FST • Corticosterone administration, GD 10–20 (in adolescence; 34)	↑ Immobility in FST • 2–3 weeks of maternal separation (1-males not studied, 132, 135)	–	–
	↑ Depression • Antepartum depression (in adolescence; 193, 198)	↑ Depression • Postpartum depression (in adolescence; 174, 202) • Antepartum depression, abuse(194)	–	–

*All data are based on outcome in adulthood unless otherwise specified. ♂ > ♀, Males more vulnerable than females; ♀ > ♂, females more vulnerable than males; ♀ = ♂, both sexes equally vulnerable; GD, gestation day*.

**Table 2:** **Effects of stress exposure at different time points throughout life on HPA axis in preclinical research (first row) and on depression in clinical research (second row)**.

**Vulnerability**	**Prenatal**	**Postnatal**	**Adolescent**	**Adult**
♂ > ♀	↑ Recovery • 3×/day, week GD 14–21 (152, 172)	↑ Peak CORT • Maternal separation 2–3 weeks (juvenile: 145; adolescent: 180)	↓ Peak CORT • Restraint, P26-33 (9)	–
	–	–	–	–
♀ > ♂	↑ Recovery • 1×/day, GD 15–19 (162) • 3×/week, GD 1–21 (264)	↓ Basal CORT • Maternal separation for 2 weeks (229) ↑ Basal CORT • Maternal separation for 3 weeks (males not studied; 1)	↓ Basal CORT • Restraint, P30-35 (in adolescence; 66) ↑ Basal CORT • Restraint, P30-52 (16)	↑ Basal CORT • Chronic mild stress (56)
	↑ Peak cortisol • Antepartum anxiety (61) • Antepartum glucocorticoids (2)	–	–	–
♀ = ♂	↑ Peak CORT • Social defeat, GD 16–20 (36)	–	↑ Peak CORT • Social instability stress, P30-45 (in adolescence; 158)	–
	–	↑ Basal CORT • Postpartum depression (99) • Abuse (106, 108)	–	–

All data are presented as outcome in adulthood unless otherwise specified. ♂ > ♀, Males more vulnerable than females; ♀ > ♂, females more vulnerable than males; ♀ = ♂, both sexes equally vulnerable; CORT, corticosterone; GD, gestation day; P, postnatal day.

**Table 3:** **Effects of stress exposure at different time points throughout life on hippocampus in preclinical research (first row) and clinical research (second row)**.

**Vulnerability**	**Prenatal**	**Postnatal**	**Adolescent**	**Adult**
♂ > ♀	–	↓ Cell proliferation • Maternal separation 2 weeks (females not studied; 170) ↓ Immature neurons • Maternal deprivation, P3 (183, 184)	↑ Cell survival • Restraint, P30-52 (16) ↑ Cell proliferation, immature neurons • Social instability, P30-45 (in adolescence; 163)	↓ Cell survival • Chronic footstock stress (265) ↓ CA3 apical dendritic neuropil • Chronic restraint stress (80) ↓ Immature neurons (dorsal) • Chronic corticosterone (33)
	–	–	–	↓ Volume • At least 2 years of depression (77)
♀ > ♂	–	↓ Immature neurons • Maternal deprivation, P3 (at weaning; 182)	↓ Cell proliferation • Restraint, P30-52 (16) • Social instability, P30-45 (in adolescence; 161)	↑ Cell survival • Chronic footstock stress (265) ↓ CA3 basal dendritic neuropil • Chronic restraint stress (80)
	–	–	–	↑ Immature neurons • No stress, with antidepressant treatment (69) ↑ Volume • No stress, with Antidepressant treatment (249)
♂ = ♀	↓ cell proliferation, survival, immature neurons • Stress during week 3 of gestation, effects throughout lifespan (20, 128, 136, 155, 207, 279)	–	–	↓ Immature neurons (ventral) • Chronic corticosterone (33)
	–	↓ Volume • Maltreatment (44)	–	–

All data are based on outcome in adulthood unless otherwise specified. ♂ > ♀, Males more vulnerable than females; ♀ > ♂, females more vulnerable than males; ♀ = ♂, both sexes equally likely to develop depression. P, postnatal day.

### Conflict of interest statement

The authors declare that the research was conducted in the absence of any commercial or financial relationships that could be construed as a potential conflict of interest.
